# Association of eHealth Literacy With Colorectal Cancer Knowledge and Screening Practice Among Internet Users in Japan

**DOI:** 10.2196/jmir.1927

**Published:** 2012-11-13

**Authors:** Seigo Mitsutake, Ai Shibata, Kaori Ishii, Koichiro Oka

**Affiliations:** ^1^Laboratory of Health and Behavioral SciencesGraduate School of Sport SciencesWaseda UniversitySaitamaJapan; ^2^Tokyo Metropolitan Institute of GerontologyTokyoJapan; ^3^Faculty of Sport SciencesWaseda UniversitySaitamaJapan

**Keywords:** eHealth Literacy, Internet, Colorectal Neoplasms, Consumer Health Information, Health Promotion

## Abstract

**Background:**

In rapidly developing Internet-user societies, eHealth literacy has become important in promoting wellness. Although previous studies have observed that poor health literacy is associated with less knowledge and screening practice of colorectal cancer (CRC), little is known about whether eHealth literacy is associated with these variables.

**Objective:**

The present study examined associations between eHealth literacy, knowledge of CRC, and CRC screening practices.

**Methods:**

Data were analyzed for 2970 Japanese adults (men, 49.9%; mean age ± SD, 39.7 ± 10.9 years) who responded to an Internet-based cross-sectional survey. Knowledge of the definition of CRC, its risk factors and screening practice, previous experience of CRC screening, score on the Japanese version of the eHEALS (J-eHEALS), sociodemographic attributes (sex, age, marital status, educational attainment, and household income level), and frequency of Internet usage were obtained. Sociodemographic attributes and frequency of Internet usage were used as control variables in the multiple regression and logistic regression models.

**Results:**

eHealth literacy was positively associated with CRC knowledge (*β* = .116, < .001), when the covariables of both eHealth literacy and CRC knowledge were used in the multiple regression model. Moreover, after controlling for sociodemographic factors, which were significantly associated with eHealth literacy and CRC screening practice, an increase of 1 point in the eHEALS score signified that participants were 1.03 times (95% CI = 1.01–1.05) more likely to undergo CRC screening.

**Conclusions:**

Internet users with high eHealth literacy are more likely to have knowledge and previous screening practice related to CRC compared to those with low eHealth literacy.

## Introduction

The Internet has become a powerful source of information for health and medicine [1,2]. Approximately 70% of Japanese Internet users seek health information on the Internet, with similar estimates reported from the United States [1,3]. Despite the proliferation of health information on websites, a critical issue has emerged—many websites purporting to provide health information are invalid or difficult to understand for individuals with low health literacy [4-7]. Previous studies have observed that limited health literacy is associated with less knowledge and poor preventive behaviors related to chronic diseases [8-10]. Because of the rapid increase in electronic health information resources, it is important that consumers improve their health literacy, and additional methods need to be developed with regard to health care and its promotion in an electronic world. These electronic health tools provide few benefits for a person’s health without “eHealth literacy,” which is an individual-level factor [11]. eHealth literacy is an individual-level factor defined as “the ability to seek, find, understand, and appraise health information from electronic sources and apply the knowledge gained to addressing or solving a health problem” [11]. eHealth literacy comprises six core skills or areas of literacy as presented in the original Lily model: (1) traditional literacy and numeracy, (2) health, (3) information, (4) science, (5) media, and (6) computer literacy [11].

Colorectal cancer (CRC) is the third most common cause of cancer death in the Japanese population, although it is largely preventable [12]. Screening tests such as the fecal occult blood test (FOBT) can reduce the morbidity and mortality of CRC [13-15]. Although the Basic Plan to Promote Cancer Control Programs has aimed to increase cancer screening rates to 50% or higher [16], only 27% of Japanese people aged >40 years have undergone CRC screening tests such as FOBT [12]. Considering the high number of Internet users who seek health information in the community, the Internet may be a key channel for disseminating information about CRC to the general population [17-20]. However, because many websites relating to CRC information are linked to commercial goods or private health services [6], consumers who use such websites without adequate eHealth literacy may purchase inappropriate goods or pay for private health services (eg, purchasing weight-loss pills with unfounded merits and paying for unnecessary services) that are actually detrimental to their health. Moreover, although much of the Internet information about CRC has been reported as being too difficult to understand for people with low health literacy [21], little is known about how eHealth literacy is associated with CRC information obtained from the Internet.

Three studies in the US showed that low health literacy was associated with less knowledge and screening practices of CRC [22-24]. In addition, people with low health literacy were less likely to seek or understand the information found about CRC from various information resources [19]. The Internet has notably become an important source of health information in Japan; this is because the Internet has gained in popularity among ordinary people through the widespread use of personal computers and cell phones [3,21,25]. Therefore, it is necessary to determine whether eHealth literacy may be useful to increase society’s awareness of CRC. Because eHealth literacy is multidimensional, it is important to begin to understand whether dimensions of eHealth literacy [26], separate and distinct from health literacy, are associated with knowledge and screening practices of CRC. However, little is known about the association between eHealth literacy and knowledge and screening practices of CRC. The present study examined this association between eHealth literacy and the knowledge and screening practices of CRC.

Previous studies of eHealth literacy have focused on particular populations: older people [27], college students [28-30], health care students [28], HIV-positive individuals [31], and parents whose children have life-threatening illnesses [32,33]. These studies used the eHealth Literacy Scale (eHEALS), a simple self-assessment scale for measuring perceived eHealth literacy [27-29,31-33]. The eHEALS was designed to be easy to use and was put through a rigorous testing process to explore the internal consistency, reliability, and validity of the instrument [29]. Vaart et al and Xie used the performance test to measure eHealth literacy [27,34]. Further, Chan et al proposed the methodological framework of eHealth literacy by characterizing complexity of eHealth tasks [35]. However, previous studies have indicated that the eHEALS does not measure all the dimensions of the Lily model of eHealth literacy [26,34]. Moreover, Vaart et al indicated that the validity of the eHEALS was insufficient because of the weak correlation between eHEALS and actual Internet use in searching for health information [34]. However, the eHEALS would appear to be more appropriate for a large-sample Internet-based survey than the performance test. It was believed to be suitable for conducting the Internet-based survey in the present study because of the eHealth literacy needed for Internet users. Therefore, in the present study, eHealth literacy was treated as perceived eHealth literacy because eHEALS was used to assess eHealth literacy [26,27,29].

## Methods

### Participants

Participants were recruited in 2009 from registrants of a Japanese Internet research service company and asked to answer a cross-sectional Internet-based survey. The research company had approximately 1,150,000 voluntarily registered participants and obtained detailed sociodemographic data (eg, sex, age, marital status, educational attainment, household income level) from each participant at the time of registration. The survey requirement in the present study was to collect data from 3000 men and women aged 20–59 years.

To remove selection bias caused by the proportional differences between gender or age group, the participants were classified by gender and placed into four age groups (20–29, 30–39, 40–49, and 50–59 years), and they were allocated equally to the eight sample groups (for each group, n = 375). Potential respondents (n = 12,435) were randomly and blindly invited from the registered samples in accordance with the set sample size and attributes, and they were invited to participate in the survey via email. The number of potential respondents in each stratified sample group was determined by dividing the quota (n = 375) by the response rate for the corresponding sociodemographic group. This rate was computed from the results of numerous previous surveys conducted by the research company (eg, for potential male respondents aged 40–49 years, the quota was 375/mean response rate of 35% = 1072). Internet-based questionnaires were placed in a protected area of a website, and the potential respondents received a specific URL in their invitation email. Potential respondents could log on to the protected area of the website using their unique log-on ID and password. After 375 participants in each group had voluntarily signed an online informed consent form, which had been approved by the institutional review board, and completed the sociodemographic data information form, acceptance of further participants was stopped in each group. The response rate of the total sample was 24.1% (3000/12,435). In addition, to remove the influence of CRC diagnosis, 14 participants diagnosed with the condition were excluded from the analyses. The present study received prior approval from the Ethics Committee of the Faculty of Sports Sciences, Waseda University, Japan.

### Measurements

#### Sociodemographic Attributes

The research company provided data on sex, age, marital status, educational attainment, and household income level. These data were collected following the participants’ registration with the research company. The participants were asked to select the category that best described their current condition for sex (male, female), age group (20–29, 30–39, 40–49, 50–59), marital status (not married, married), education level (graduate school, college, two-year college, career college, high school, junior high school), and household income (<3 million yen [about US$37,500], 3–5 million yen [about US$37,500−US$62,500], 5–7 million yen [about US$62,500–US$87,500], 7–10 million yen [about US$87,500−US$125,000], and ≥10 million yen [about US$125,000]). Moreover, since few participants answered the questions on graduate school, two-year college, career college, and junior high school, the category groups for education level were divided into the following three categories: ≤ high school graduate (high school and junior high school); two-year college or career college (two-year college and career college); and ≥ college graduate (graduate school and college).

#### Frequency of Internet Searching

Daily frequency of information searches on the Internet was assessed by the following four response categories: Every day, 4–5 times/week, 2–3 times/week, or ≤1 time/week.

#### eHealth Literacy

The Japanese version of eHEALS (J-eHEALS) was used to assess eHealth literacy levels of participants [3]. J-eHEALS uses a 5-point Likert scale (ranging from 1, strongly disagree, to 5, strongly agree; score range, 8-40) to measure perceived eHealth literacy for participants. To determine the validity of J-eHEALS, confirmatory factor analysis was conducted using the data from administration of the present survey. This analysis for the eight-item model suggested a good fit for the proposed model (GFI = .988, CFI = .993, RMSEA = .056). In addition, the internal reliability of the test was confirmed using Cronbach alpha coefficient = 0.93 (*P* < .01).

#### CRC Knowledge Test

Knowledge of CRC was assessed by 20 true/false questions regarding the definition, risk factors, and screening of CRC. This self-administered test was adapted from previous studies of knowledge and attitudes of CRC [36,37]. The true/false instrument score ranged from 0 (low) to 20 (high).

#### Previous CRC Screening Practice

The participants were asked whether they had ever undergone CRC screening by answering “Yes” or “No”.

### Statistical Analyses

Data were analyzed for 2970 adults (response rate: 24.1%) who provided complete information for the study variables. Patients excluded from the data analysis included 16 participants with incomplete information and 14 participants diagnosed with CRC. The *t* test was used to examine the differences in eHEALS score and CRC knowledge score between male and female and between married and unmarried individuals. In addition, the differences in eHEALS score and CRC knowledge score among three or more category groups (age, education level, household income level, and frequency of Internet searching) were examined using one-way ANOVA. Moreover, a chi-square test was employed to evaluate the proportional differences in CRC screening practice for sociodemographic variables and frequency of Internet searching. In accordance with the analytical methodology adopted in previous studies of health literacy and CRC knowledge and practice [23,24,38], the variables of sociodemographic attributes and frequency of Internet searching (which achieve statistical significance in association with CRC knowledge score and CRC screening practice from bivariate analyses) were included in the multiple regression and logistic regression models as covariates. Subsequently, multiple regression analyses adjusted for these covariates were conducted to examine the association between eHealth literacy and CRC knowledge. Moreover, we performed logistic regression analyses adjusted for these covariates to assess the impact of eHealth literacy on CRC screening practice. Additionally, *P* < .05 was considered statistically significant in all analyses. Adjusted ORs and 95% confidence intervals (CI) were calculated for each variable. PASW Statistics 18.0 was used to compute the statistics.

## Results

### Characteristics of Respondents

Table 1 presents characteristics of the respondents. In the current study, the mean age (SD) of study participants was 39.7 (10.9) years, and 49.9% (n = 1483) of the participants were males. Overall, 60.9% (n = 1809) of the respondents were married, approximately 51.6% (n = 1534) had graduated from college or graduate school, and 23.6% (n = 702) were educated to a level below a high school diploma. Of the respondents, 17.4% (n = 516) had a household income <3 million yen (about US$37,500) and 13.3% (n = 396) earned >10 million yen (about US$125,000). Seventy percent (n = 2086) of respondents used the Internet every day. The mean J-eHEALS score was 23.5 (SD = 6.5). Overall, 58.9% (n = 1748) had high eHealth literacy and 41.1% (n = 1222) of the respondents had a low eHealth literacy level. The mean CRC knowledge test score was 13.8 (SD=2.4). Approximately 59.5% (n = 1766) had a high level of knowledge about CRC, and 19.7% (n = 584) had previously undergone CRC screening.

### Association Between eHealth Literacy and CRC Knowledge Adjusted for Covariates

Table 2 presents the differences in eHEALS score and CRC knowledge score with sex and marital status using the *t* test. Also, Table 3 shows the differences in eHEALS score and CRC knowledge score with age group, education level, household income, and frequency of Internet searching using the one-way ANOVA. In bivariate analyses, education level was not statistically significantly related to eHealth literacy (*P* = .07). Moreover, education level (*P* = .136) and frequency of Internet searching (*P* = .08) were not statistically significantly related to CRC knowledge test score. Since sex, age group, marital status, and household income were statistically associated with both eHealth literacy level and CRC knowledge level, these variables were included in the multiple regression model as controlling factors.

Table 4 presents the results of the multiple regression analyses for the association between eHealth literacy and CRC knowledge after controlling for sex, age group, marital status, and household income. The regression model was significant and accounted for 4.6% of the CRC knowledge (R = .221, adjusted R^2^ = .046, *P* < .001). When all the controlled variables were entered into the regression model, eHealth literacy was found to be positively associated with CRC knowledge (*β* = .116, s*tructure coefficient* = .602). In addition, all the controlled variables were significant contributors to the knowledge score of CRC. Moreover, age was a stronger contributor than the other controlled variables.

### Association Between eHealth Literacy and CRC Screening Practice Adjusted for Covariates

Table 5 presents the differences of CRC screening practice with sociodemographic characteristics and frequency of Internet searching using the chi-square test. From the sample, the chi-square test indicated that sex (*P* = .38) and frequency of Internet searching (*P* = .173) were not related to CRC screening practice. By contrast, participants who had undergone CRC screening were more likely to be older adults (*P* < .001), be married (*P* < .001), have a higher education level (*P* = .03), and have higher household income (*P* < .001) than reference groups.

Table 6 presents the results of the logistic regression for the association between eHealth literacy and the CRC screening practice after controlling for age, marital status, education level, and household income level. After controlling for these factors, an increase in the eHEALS score by 1 point signified that the subjects of the present study were 1.03 times (95% CI = 1.01–1.05) more likely to undergo CRC screening.

**Table 1:** Sociodemographic characteristics (numbers and percentages).

Characteristics		n	%
**Sex**			
	Male	1483	49.9
	Female	1487	50.1
**Age group**			
	20–29	739	24.9
	30–39	746	25.1
	40–49	742	25
	50–59	743	25
**Marital status**			
	Not married	1161	39.1
	Married	1809	60.9
**Education level**			
	≤ High school graduate	702	23.6
	2-year college or career college	734	24.7
	≥ College graduate	1534	51.6
**Household income (yen)** ^**a**^			
	<3 million	516	17.4
	3–5 million	838	28.2
	5–7 million	620	20.9
	7–10 million	600	20.2
	>10 million	396	13.3
**Frequency of Internet searching (per week)**			
	Every day	2086	70.2
	4–5 times	374	12.6
	2–3 times	248	8.4
	≤1 time	262	8.8
**eHealth literacy level** ****			
	High eHealth literacy (≥24)	1748	58.9
	Low eHealth literacy (<24)	1222	41.1
**Knowledge of CRC**			
	High (≤14)	1766	59.5
	Low (>14)	1204	40.5
**CRC screening**			
	Yes	584	19.7
	No	2386	80.3

^a^ $1 = 80yen, in 2011/12.

**Table 2:** Association of eHealth literacy and knowledge of CRC with sex and marital status (using the *t* test).

Characteristics		eHealth literacy score			CRC knowledge test score		
		Means	SD	*P* values	Means	SD	*P* values
**Sex**				.002			< .001
	Male	23.15	6.73		13.57	2.46	
	Female	23.87	6.15		14.11	2.27	
**Marital status**				.016			< .001
	Not married	23.15	6.63		13.48	2.48	
	Married	23.74	6.33		14.06	2.29	

**Table 3:** Association of eHealth literacy and knowledge of CRC with age, educational level, household income and frequency of Internet searching (using the one-way ANOVA).

Characteristics		eHealth literacy score			CRC knowledge test score		
		Means	SD	*P* values	Means	SD	*P* values
**Age group**				.01			< .001
	20–29	22.75	6.53		13.30	2.44	
	30–39	23.46	6.38		13.76	2.46	
	40–49	24.06	6.55		14.18	2.30	
	50–59	23.77	6.30		14.10	2.23	
**Education level**				.07			.136
	≤ High school graduate	23.07	6.43		13.70	2.32	
	2-year college or career college	23.44	6.48		13.95	2.32	
	≥ College graduate	23.74	6.45		13.85	2.44	
**Household income (yen)** ^**a**^							< .001
	<3 million	23.02	6.54	< .001	13.47	2.39	
	3–5 million	23.09	6.36		13.71	2.40	
	5–7 million	23.30	6.32		13.86	2.37	
	7–10 million	23.85	6.35		14.18	2.31	
	>10 million	24.84	6.73		14.03	2.38	
**Frequency of Internet searching (per week)**							
	Every day	22.35	6.50	< .001	13.50	2.46	.083
	4–5 times	22.19	6.11		13.96	2.40	
	2–3 times	22.46	6.26		13.78	2.47	
	≤1 time	24.00	6.46		13.88	2.35	

^a^ $1 = 80yen, in 2011/12.

**Table 4:** Multiple regression for Knowledge score of CRC by eHealth literacy and sociodemographic factors.

	*Β*	SE (*β*)	r_s_^a^	r^b^	*P* values
eHealth literacy	.116	.007	.602	.133	< .001
Sex (Male=1, Female=0)	-.103	.087	-.516	-.114	< .001
Age group	.083	.004	.598	.125	< .001
Marital status (Married=1, Not married=0)	.048	.102	.539	.119	.02
Household income	.046	.035	.417	.092	.02

^a ^r_s _= *structure coefficient.*

^b ^r = correlation coefficient.

**Table 5:** Association of CRC screening practice with sociodemographic characteristics and frequency of Internet searching.

Characteristics		Yes	%	No	%	*P* values
**Sex**						.38
	Male	282	48.3	1201	50.3	
	Female	302	51.7	1185	49.7	
**Age group**						< .001
	20–29	12	2.1	727	30.5	
	30–39	55	9.4	691	29.0	
	40–49	221	37.8	521	21.8	
	50–59	296	50.7	447	18.7	
**Marital status**						< .001
	Not married	107	18.3	1054	44.2	
	Married	477	81.7	1332	55.8	
**Education level**						.03
	≤ High school graduate	128	21.9	574	24.1	
	2-year college or career college	169	28.9	565	23.7	
	≥ College graduate	287	49.1	1247	52.3	
**Household income (yen)** ^**a**^						< .001
	<3 million	62	10.6	454	19.0	
	3–5 million	121	20.7	717	30.1	
	5–7 million	107	18.3	513	21.5	
	7–10 million	186	31.8	414	17.4	
	>10 million	108	18.5	288	12.1	
**Frequency of Internet searching (per week)**						.173
	Every day	411	70.4	1675	70.2	
	4–5 times	61	10.4	313	13.1	
	2–3 times	58	9.9	190	8.0	
	≤1 time	54	9.2	208	8.7	

^a^ $1 = 80yen, in 2011/12.

**Table 6:** Adjusted odds ratios for CRC screening practice by eHealth literacy level.

	Predictor *B*	(SE)	Lower	exp b	Upper	Wald X^2^	*P* values
eHealth literacy	.03	.01	1.01	1.03	1.05	11.90	.001
Age group	.10	.01	1.09	1.11	1.12	277.68	< .001
Marital status(Married=1, Not married=0)	.36	.13	1.10	1.43	1.86	7.29	.007
Education level	.04	.06	.92	1.04	1.18	.38	.539
Household income	.08	.04	1.00	1.09	1.18	3.76	.052

## Discussion

The present study is the first to examine the association between eHealth literacy and knowledge and screening practice of CRC. The present study found that high eHealth literacy was associated with high knowledge about CRC and CRC screening practice. Considering a great increase in the number of Internet users, the Internet may be an important channel for providing information about CRC screening among the general population [17-21]. Thus, adequate eHealth literacy will become an important factor in improving CRC knowledge and promoting CRC screening practice using the Internet.

The present study found that higher eHealth literacy was associated with higher knowledge and screening practices of CRC even after controlling covariables. These findings are consistent with those observed in the previous studies with respect to associations of health literacy with knowledge and practice about CRC [22-24]. However, these previous studies on health literary indicated that education level was a strong covariate of health literacy [23,24,38], whereas education level was not a statistically significant covariate of eHealth literacy in our study. In contrast, two studies in the United States and Israel about eHealth literacy assessed by eHEALS found that a lower educational level was associated with lower eHealth literacy [33,39]. The use of non-Internet users as participants in previous studies may explain the inconsistencies with the results regarding education level in the present study: registrants of an Internet research service company might have a higher education level than non-registrant Internet users and non-Internet users [3,40]. eHealth literacy is important for Internet users utilizing web-based CRC information since it is estimated that the number of Internet users continues to increase regardless of sociodemographic factors, such as age and education level [3,41]. Therefore, more studies are apparently needed among Internet users to clarify the role of education level and other sociodemographic factors as covariates of eHealth literacy [3,26,33,41].

Since the present study demonstrates the positive association between eHealth literacy and the knowledge about CRC and previous CRC screening practice, both designing the CRC information websites for those with low eHealth literacy levels and developing an intervention for enhancing eHealth literacy might be required strategies in order to improve the knowledge and enhance screening practice of CRC. First, the previous studies found that CRC information websites were often too difficult for American adults with limited literacy to use and understand [6,21]. For one third of Americans with limited health literacy, this may pose a problem in using Internet-based CRC information. Therefore, Friedman et al suggested that health professionals, health informaticians, medical journalists, and web page editors must collaborate to ensure the use of plain language to match the literacy skills of consumers [21]. Also, a Japanese study reported that even the most prominent cancer information website needed to improve its usability and readability to provide cancer information effectively [20]. Thus, the websites of CRC information should be designed to match the low eHealth literacy levels of target populations and incorporate video, graphics, animation, and audio narratives using easy-to-understand language [17,21]. Secondly, an intervention to improve eHealth literacy should be developed in order to use eHealth information effectively to modify health behavior. Recent studies suggested interventions to improve eHealth literacy for older adults and HIV-positive patients with low eHealth literacy [27,31]. These studies found that educational interventions for basic knowledge and skills in using the Internet and evaluating online health information significantly improved eHealth literacy among populations with low eHealth literacy [27,31]. The results of the present cross-sectional Internet-based survey indicate that Internet users with low eHealth literacy have less knowledge about CRC and are less likely to undergo CRC screening. A future intervention study should therefore examine whether improving eHealth literacy through educational programs can enhance knowledge about CRC and promote CRC screening behavior among Internet users with low eHealth literacy.

Future studies should identify subcomponents of eHealth literacy such as specific skills or health literacy among at-risk subgroups, in order to design interventions that improve eHealth literacy in Japan. For example, although people in Japan with higher frequency of Internet searching have high eHealth literacy, young adults who use the Internet more than 30−60 minutes have lower eHealth literacy level than older adults [40]. This suggested that low eHealth literacy of young adults may be influenced by multidimensional literacy of eHealth literacy without computer literacy. However, it is unclear how lower eHealth literacy of young adults might be influenced by subcomponents of eHealth literacy. Moreover, in the previous studies mentioned, it is problematic that eHEALS focuses on only one or two aspects of the Lily Model of eHealth literacy [26,34]. Therefore, future studies need to assess each of the six literacies in the Lily Model to consider the influence of sub-dimensions on eHealth literacy [26].

### Limitations

The present investigation has some limitations. First, the analysis was cross-sectional, thereby making determinations of cause and effect not feasible. Second, participants were recruited from one Japanese Internet research service company, and thus the relationships assessed may be biased because of the potentially nonrepresentative nature of this sample as general Japanese Internet users [42]. Also, because the registrants of the research service company were frequent Internet users, the participants of the present study may be skewed toward a high eHealth literacy level. Moreover, since the present study indicated the low effect sizes of the multiple and logistic regressions, the statistical significance from the results of the multivariable analysis in the present study might be an artifact of a large sample size.

### Conclusions

Among Japanese adult Internet users, individuals with low eHealth literacy have less knowledge about CRC and are less likely to undergo CRC screening practice. To promote information about CRC screening on the Internet for individuals who need to undergo CRC screening, it is important to improve eHealth literacy among the appropriate populations. In addition, it is essential to design websites containing CRC information specifically for those with low eHealth literacy.
